# Characterization of the core microbiota of the drainage and surrounding soil of a Brazilian copper mine

**DOI:** 10.1590/S1415-475738420150025

**Published:** 2015

**Authors:** Letícia Bianca Pereira, Renato Vicentini, Laura M.M. Ottoboni

**Affiliations:** Centro de Biologia Molecular e Engenharia Genética, Universidade Estadual de Campinas, Campinas, SP, Brazil

**Keywords:** mine drainage, soil, 16S rDNA pyrosequencing, core microbiota, generalist and specialist OTUs

## Abstract

The core microbiota of a neutral mine drainage and the surrounding high heavy metal content soil at a Brazilian copper mine were characterized by 16S rDNA pyrosequencing. The core microbiota of the drainage was dominated by the generalist genus *Meiothermus*. The soil samples contained a more heterogeneous bacterial community, with the presence of both generalist and specialist bacteria. Both environments supported mainly heterotrophic bacteria, including organisms resistant to heavy metals, although many of the bacterial groups identified remain poorly characterized. The results contribute to the understanding of bacterial communities in soils impacted by neutral mine drainage, for which information is scarce, and demonstrate that heavy metals can play an important role in shaping the microbial communities in mine environments.

Microbial communities are strongly influenced by environmental factors that determine the activity and spatial dynamics of microorganisms in natural or anthropic environments. Information about the structure and behavior of the bacterial communities in a particular environment can be obtained from identification of the generalist and specialist bacteria present. For example, Barberán *et al*. (2011) used 16S rDNA pyrosequencing to define the specialist and generalist OTUs (operational taxonomic units) of 151 soil samples from locations throughout the American continent and Antarctica, and observed that generalist OTUs were formed by bacterial groups common to soils, such as *Acidobacteria, Proteobacteria*, and *Verrucomicrobia*. In contrast, specialist OTUs were more often found in extreme environments, such as desert and Antarctica, and consisted of taxa that are less common in soils, such as *Chloroflexi* and *Deinococcus*.

Other information that is important for understanding the structure of a microbial community is knowledge of the core microbiota. The core microbiota can be defined as a group of microorganisms common to two or more habitats (Turnbaugh *et al*., 2007; Hamady and Knight, 2009). The identification of the core microbiota of a given environment can assist in ecological studies, because the occurrence of the same microorganisms in all samples suggests that they could play a crucial role in the maintenance of the community. Studies of the core microbiota can also help in prediction of the response of the microbial community to environmental disturbances (Shade and Handelsman, 2012). Sun *et al*. (2013) evaluated the diversity of bacteria in six estuaries with different levels of contamination by polycyclic aromatic hydrocarbons (PAHs) and heavy metals. The results showed that ten OTUs had significant correlations with environmental and contaminant variables, and it was concluded that the core microbiota was more affected by environmental and contamination variations, compared to other bacteria.

Neutral mine drainage is a common environmental disturbance in mining areas. It is an aqueous solution derived from the oxidation of minerals, and is generally rich in calcium, sodium, magnesium, and heavy metals (Rose and Cravotta, 1998; Lottermoser, 2007). However, neutral mine drainage environments remain poorly explored, and little is known about the structures and diversity of the bacterial communities present (Liao and Xie, 2007; Guo *et al*., 2009). In this work, a 16S rDNA pyrosequencing analysis was performed to characterize the core microbiota of a neutral mine drainage and the surrounding high heavy metal content soil, in order to obtain new insights into microbial ecology in environments contaminated with heavy metals.

The procedures employed for sample collection, pyrosequencing of the 16S rDNA, data analysis, and chemical analysis have been described in detail in Pereira *et al*. (2014). Briefly, six drainage samples (D1-6) and six soil samples (S1-6, from points adjacent to the drainage sample sites) were collected. The DNA was isolated, and the V3-V4 region of the 16S rDNA was amplified using the 338F and 806R primers (Huse *et al*., 2008; Masoud *et al*., 2011). The amplicons were sequenced with the 454 GS Junior platform (Roche, Branford, CT, USA) and the pyrosequencing data were submitted to MG-RAST (Meyer *et al*., 2008) (IDs 4521082.3 to 4521090.3, and 4521341.3 to 4521343.3). Sixteen chemical parameters (cadmium, calcium, lead, copper, chromium, sulfur, iron, phosphorus, magnesium, manganese, nickel, potassium, sodium, zinc, pH, and organic matter) were also determined for each sample (Environmental Protection Agency, 1986; Camargo *et al*., 2009). Evaluation of the quality of the sequences (quality score ≥ 25 and size between 400 and 470 bp) and their grouping into OTUs (similarity of 97% or more) was performed with the QIIME package (Caporaso *et al*., 2010), and taxonomic classification was performed using the Ribosomal Database Project (RDP, Maidak *et al*., 1996), with a threshold of 80%.

A null model based on the Bray-Curtis β-diversity index was performed to determine if the mine community was shaped by a stochastic or a deterministic process, as described by Zhou *et al*. (2013). Briefly, a permutational analysis of multivariate dispersions (PERMDISP) was used to test the difference between the β-diversity in the community and in the null model. This analysis was performed using the vegan (Oksanen *et al*., 2013) package for R (R Development Core Team, 2013). The phylogenetic tree of the 50 most abundant OTUs was constructed using the Tamura-Nei method (with 1000 bootstrap replicates), performed with MEGA v. 3.1 software (Kumar *et al*., 1993).

Graphical representations of the distribution and abundance of OTUs in the samples were used to identify generalist and specialist OTUs. Generalist OTUs were considered those that presented high relative abundance (≥ 2% of the mean abundance, represented by the outliers in the graph) in all samples of drainage or soil. Specialist OTUs were those that appeared in high abundance (≥ 2% of the mean abundance, represented by the outliers in the graph) in one or two samples from each environment. Spearman rank correlation analyses were used to correlate the chemical parameters with the specialist and generalist OTUs (p < 0.05). The core microbiota consisted of the OTUs that were present in all the soil or drainage samples.

A total of 5,901 OTUs were obtained from high-throughput 16S rDNA pyrosequencing of the six drainage samples and six soil samples (Pereira *et al*., 2014). The PERMDISP analysis revealed that there was no significant difference between the β-diversity of the mine community and the β-diversity expected based on the null model (F = 3.65, p-value = 0.08). These results suggested that the bacterial community in the Sossego copper mine was influenced by stochastic factors. Pereira *et al*. (2014) described the importance of several chemical parameters that influenced the composition of the microbial community samples from this mine. However, the non-significant p-value obtained with the PERMDISP analysis could also be indicative of an interaction between stochastic and deterministic factors. This kind of interaction between the factors has been described previously for different environments (Stegen *et al*., 2012; Zhou *et al*., 2013; Valverde *et al*., 2014). Therefore, it is likely that the Sossego copper mine bacterial community was driven by both factors (stochastic and deterministic).

The phylogenetic tree (Figure 1) of the most abundant OTUs showed that the mine environment was dominated by the phylum *Proteobacteria* (especially *Alphaproteobacteria*, followed by *Betaproteobacteria, Gammaproteobacteria*, and *Deltaproteobacteria*), *Acidobacteria, Gemmatimonaedetes, Actinobacteria*, and *Deinococcus/Thermus*. Many unclassified bacteria were also observed in high abundance. The phylum *Proteobacteria*, with six different families, showed the greatest number of identified families.

**Figure 1:**
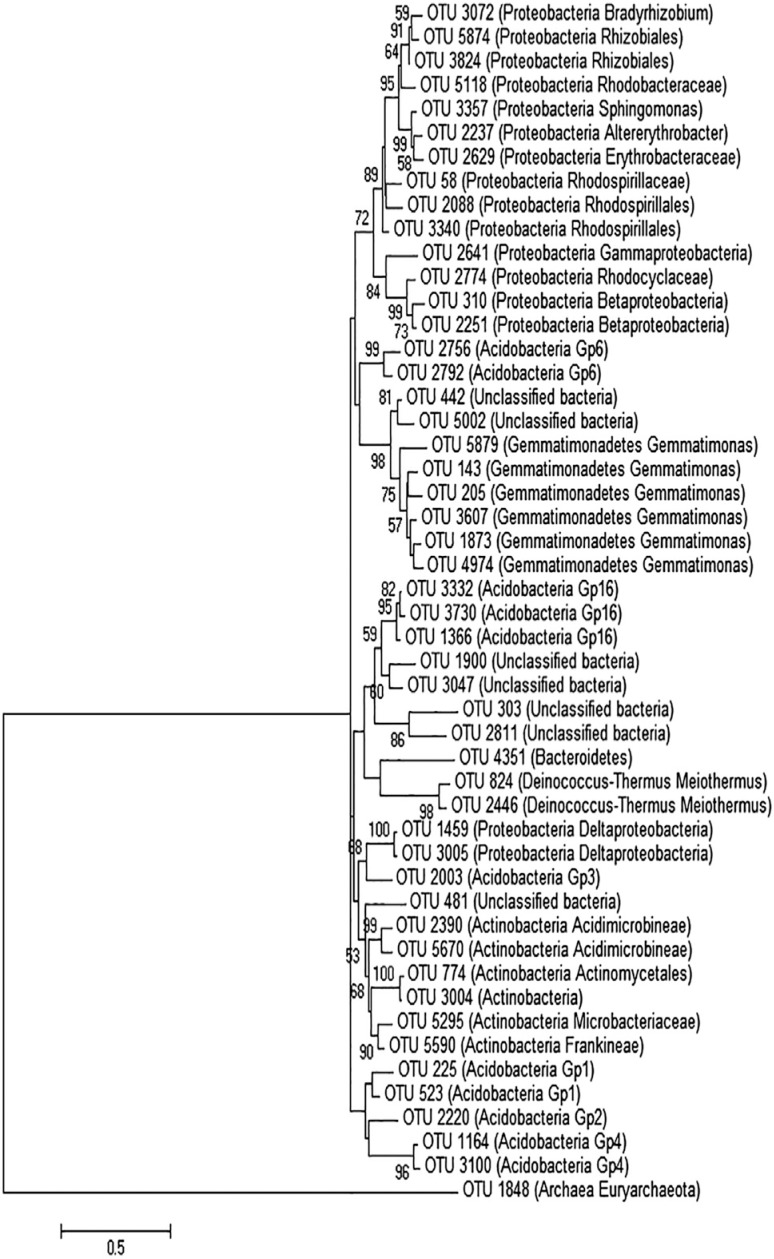
Phylogenetic tree based on the partial 16S rRNA gene sequences from the 50 most abundant OTUs present in the Sossego mine. Bootstrap values (1,000 replicates) higher than 50% are shown.

For the determination of habitat generalists and specialists, each OTU was plotted in graphs of mean abundance (on a logarithmic scale) against number of samples (Figure 2A,B, for the drainage and soil samples, respectively), and the Spearman rank correlation test was used to correlate the chemical parameters (Pereira *et al*., 2014) with the OTUs (Table S1, Supplementary Material).

**Figure 2:**
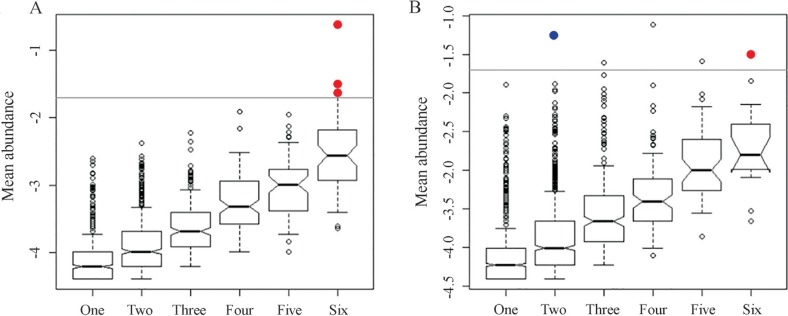
Box plots showing the occupancy and abundance of OTUs in the drainage (A) and soil (B) samples. Specialist OTUs (outliers present in one or two samples in high abundance) are shown in blue. Generalist OTUs (outliers present in all samples in high abundance) are shown in red. The lines in the graphs represent 2% relative abundance of the OTUs.

The neutral drainage environment presented no specialist OTUs and three OTUs that were considered generalists. The latter OTUs were 824 (*Meiothermus*), 1366 (*Acidobacteria*), and 3607 (*Gemmatimonas*). The OTU 824 presented the highest relative abundance in all the drainage samples (data not shown), and was strongly correlated with copper (0.77), nickel (0.84), and sulfur (0.88). The correlation with these chemical parameters suggested that this OTU was able to grow in the presence of high concentrations of heavy metals, as found in the drainage environment, and this ability therefore explained the existence of this OTU in all the drainage samples. *Meiothermus* is a bacterial genus that is generally associated with areas affected by anthropogenic activity, and was the principal taxon found in neutral mine drainage of the Sossego mine (Pereira *et al*., 2014).

The generalist OTU 1366 was classified as *Acidobacteria* and showed a strong correlation with sulfur (0.82). Very little is known about the ecological role of this phylum, although it has been found in a wide range of environments including soils, sediments, thermal waters, soils contaminated with heavy metals, and mine drainages (Rappe and Giovannoni, 2003; Thrash and Coates, 2010). The OTU 3607 was correlated with sulfur (0.84). This genus has been reported in different mine environments including soil and sediments contaminated with heavy metals (Mendez *et al*., 2008; Reis *et al*., 2013).

High levels of sulfur were found in the drainage samples, which could account for the strong correlation observed between this chemical parameter and the generalist OTUs. Although very little is known about these OTUs, it is possible that they could participate in the sulfur cycle, enabling them to grow in all the drainage samples. The absence of specialist OTUs in the drainage samples suggests that this environment had few different niches that could be occupied, and/or little interspecies competition for resources within the community.

The specialist OTU 303 in the soil samples could not be classified into any taxon. The generalist OTU 310 was identified as a bacterium belonging to the suborder *Frankinae* and was correlated with sulfur (0.82).

Despite the fact that neither the generalist nor the specialist OTUs provided any important information about the soil community, the fact that specialist OTUs were present in the soil habitat suggests the existence of a more heterogeneous community, in terms of niches, compared to the drainage community. Moreover, the soil community could provide a higher level of interspecies competition for resources, resulting in bacterial groups that were better adapted to certain niches.

The core microbiota was defined by OTUs that were present in all the drainage or soil samples. In the drainage environment, 60 OTUs were present in all the samples collected along the channel (Table S2, Supplementary Material), while in the soil samples the core microbiota was composed of 17 OTUs (Table S3, Supplementary Material). The smaller number of OTUs found in all the soil samples suggests that the soil was a more heterogeneous environment, in terms of occupied niches, compared to the drainage. A more heterogeneous environment is also consistent with the appearance of specialist OTUs.

Evaluation of the taxa in the core microbiota enabled the discrimination of several general groups.

(i) Bacteria resistant to heavy metals: In the drainage samples, there were a large number of OTUs related to bacteria generally resistant to heavy metals (especially copper), including the genera *Bradyrhizobium, Sphingomonas*, and *Meiothermus*. The genus *Meiothermus* was the most abundant bacterial group in the core microbiota of the drainage (48.6% of the sequences), and was recently reported to be present in the Sossego copper mine (Pereira *et al*., 2014). The genus *Bradyrhizobium* includes bacteria capable of fixing atmospheric nitrogen in association with plant root nodules, and these organisms are usually tolerant of heavy metals (Jordan, 1982; Garrity and Holt, 2001; Sadowsky and Graham, 2006). *Sphingomonas* is known to be associated with the corrosion of copper pipes and the biosorption of copper (White *et al*., 1996). Vilchez *et al*. (2007) studied the efficiency of a biofilm filter for the removal of copper from groundwater. The metal removal efficiency reached 90%, and organisms from the genus *Sphingomonas* were predominant in the biofilms (78% of the sequences analyzed). Wang *et al*. (2010) investigated the absorption of copper by the bacterium *Sphingomonas paucimobilis*, as well as its potential for the treatment of effluents containing high levels of heavy metals. Altimira *et al*. (2012) used pyrosequencing to study bacterial diversity in agricultural soils contaminated by copper in Chile. The fact that bacteria from the genus *Sphingomonas* contained copper resistance genes in plasmids could contribute to the distribution of these genes in the soil bacterial community.

In the soil samples, the genus *Bradyrhizobium* was also found in the core microbiota. The genus *Blastococcus* was only found in the core microbiota of the soils. The physiology of this genus remains poorly characterized, but studies have shown that this group of bacteria is more resistant to heavy metals compared to other related groups, and can use a variety of carbon sources, which provides an explanation for its presence in all the soil samples (Essoussi *et al*., 2010; Chouaia *et al*., 2012).

(ii) Xenobiotic-degrading bacteria: No taxa associated with the degradation of xenobiotic compounds were found in the core microbiota of the soil. However, the drainage samples contained several taxa associated with xenobiotic degradation, identified as *Phenylobacterium, Porphyrobacter, Novosphingobium*, and the *Rhodocyclaceae* family. The genus *Phenylobacterium* has important biotechnological potential, because it includes species capable of using xenobiotic compounds (such as chloridazon and pyramidon herbicides) as carbon sources (Eberspächer and Lingens, 2006). The genus *Porphyrobacter* includes members that are able to degrade aromatic compounds such as biphenyl and dibenzofuran (Hiraishi *et al*., 2002). The genus *Novosphingobium* contains species capable of metabolizing contaminants such as polychlorophenol (Tiirola *et al*., 2002), aromatic compounds (Liu *et al*., 2005), and estradiol (Fujii *et al*., 2003). The *Rhodocyclaceae* family has been identified in many studies of the bacterial diversity of soil contaminated with xenobiotic compounds. Singleton *et al*. (2011) used 16S rDNA pyrosequencing to analyze bacterial diversity in bioreactors used to treat soil contaminated with polycyclic aromatic hydrocarbons and found sequences associated with the *Rhodocyclaceae* family during degradation of these compounds. Martin *et al*. (2012) evaluated bacterial diversity in soil contaminated with phenanthrene and found a predominance of *Betaproteobacteria*, including organisms belonging to the *Rhodocyclaceae* family, which were related to the mineralization of phenanthrene.

(iii) Poorly characterized bacterial groups: Many bacterial groups of the core microbiota of the Sossego mine have been poorly characterized or have only recently been discovered, so little is known about their ecology and applicability. An abundant group found in the drainage samples was the genus Gemmatimonas, which is often found in soil and mine environments (Mendez *et al*., 2008; Reis *et al*., 2013). The core community of the drainage also included sequences associated with the suborder *Nannocystineae*, the phylum *Acidobacteria*, the families *Microbacteriaceae* and *Geodermatophilaceae*, and the genera *Flavisolibacter* and *Ohtaekwangia*. In the soil samples, sequences classified as belonging to the phyla *Alphaproteobacteria, Betaproteobacteria*, and *Actinobacteria*, as well as the suborder *Acidimicrobineae*, and the order *Actinomycetales* were found.

The orders S*phingomonadales* and *Rhizobiales*, the genus *Bradyrhizobium*, and the phylum *Acidobacteria* were found in both the soil and the drainage environments. As mentioned previously, these bacteria are common in soils and present important characteristics such as nitrogen fixation and resistance to heavy metals. These taxa could play an important role in the Sossego mine soil, because they were included in the core microbiota of the two environments. In an earlier work, Golebiewski *et al*. (2014) analyzed the bacterial diversity of soils contaminated with heavy metals in the area surrounding a zinc and lead enrichment plant. Upon evaluation of the core microbiota of this environment they found sequences associated with the genus *Sphingomonas*, the family *Acidobacteriaceae*, the orders *Rhodospirillales* and *Rhizobiales*, and the phyla *Acidobacteria* and *Chloroflexi*. Sequences corresponding to these taxa were also found for the microbiota present in the drainage and soil of the Sossego mine, suggesting that they could play key roles in the bacterial communities of environments contaminated with heavy metals, especially in soils with neutral pH.

In conclusion, the distribution of OTUs, together with core microbiota analysis, indicated that in terms of niches the drainage environment was relatively homogeneous, while the soil environment was more heterogeneous. The core microbiota of the Sossego mine generally consisted of heterotrophic bacteria with optimum growth at neutral to alkaline pH and resistance to heavy metals. The core microbiota from the drainage included bacteria with potential biotechnological applications for the degradation of xenobiotic compounds. This work now provides new information about the core microbiota in neutral mine drainage and soil environments, showing that the majority of the bacteria in this kind of environment remain unknown or poorly characterized. These organisms could offer many possibilities for studies leading to novel industrial and biotechnological applications.

## Supplementary Material

The following online material is available for this article:

Table S1 Spearman rank correlation results.

Table S2 - OTUs present in all drainage samples, and their taxonomic classification in the RDP database

Table S3 - OTUs present in all soil samples, and their taxonomic classification in the RDP database

This material is available as part of the online article from: http://www.scielo.br/gmb.
