# Supplementary material for: Characterization of the core microbiota of the drainage and surrounding soil of a Brazilian copper mine
Source: Genet Mol Biol. 2015 Oct-Dec;38(4):484–9. doi: 10.1590/S1415-475738420150025 (PMC4763313; doi:10.1590/S1415-475738420150025)
Supplement: Table S1 [file 1415-4757-gmb-S1415-475738420150025-s001.pdf]

Table S1. Spearman rank correlation results.

	824	1366	310	303
**Cd**	-0.232	-0.406	0.588	0.191
**Ca**	-0.143	-0.486	0.314	-0.676
**Pb**	-0.314	-0.257	0.371	0.541
**Cu**	0.771*	0.600	0.486	0.541
**Cr**	0.086	0.143	0.429	0.068
**S**	0.886*	0.829*	0.829*	-0.169
**Fe**	-0.143	-0.029	0.714	-0.169
**P**	0.200	0.543	0.086	0.541
**Mg**	0.406	0.377	-0.086	-0.372
**Mn**	0.429	0.143	0.771	-0.169
**Ni**	0.841*	0.522	0.143	0.541
**K**	0.086	0.371	-0.086	-0.845
**Na**	0.029	-0.087	-0.200	-0.034
**Zn**	0.086	-0.029	-0.029	0.541
**pH**	-0.319	-0.319	0.029	-0.845
**MO**	0.754	0.609	0.657	-0.338

* Statistically significant at 5%.
