# Supplementary material for: Characterization of the core microbiota of the drainage and surrounding soil of a Brazilian copper mine
Source: Genet Mol Biol. 2015 Oct-Dec;38(4):484–9. doi: 10.1590/S1415-475738420150025 (PMC4763313; doi:10.1590/S1415-475738420150025)
Supplement: Table S2 - [file 1415-4757-gmb-S1415-475738420150025-s002.pdf]

Table S2. Core microbiota: OTUs present in all drainage samples, and their taxonomic classification in the RDP database. The minimum cuttof value is 80%.

	RDP classifier [bootstrap]					
**OTU**	**Domain**	**Phylum**	**Class**	**Order**	**Family**	**Genus**
**58**	Bacteria [100%]	Proteobacteria [100%]	Alphaproteobacteria [100%]	Rhodospirillales [97%]	Rhodospirillaceae [92%]	—
**76**	Bacteria [100%]	Proteobacteria [100%]	Alphaproteobacteria [96%]	—	—	—
**104**	Bacteria [100%]	Proteobacteria [100%]	Alphaproteobacteria [100%]	Rhizobiales [100%]	—	—
**205**	Bacteria [100%]	Gemmatimonadetes [100%]	Gemmatimonadetes [100%]	Gemmatimonadales [100%]	Gemmatimonadaceae [100%]	Gemmatimonas [100%]
**310**	Bacteria [100%]	Proteobacteria [100%]	Betaproteobacteria [97%]	—	—	—
**410**	Bacteria [100%]	Proteobacteria [100%]	Alphaproteobacteria [100%]	Sphingomonadales [100%]	Erythrobacteraceae [99%]	Porphyrobacter [99%]

**442**	Bacteria [100%]	—	—	—	—	—
**631**	Bacteria [100%]	Actinobacteria [96%]	Actinobacteria [96%]	Actinomycetales [87%]	—	—
**824**	Bacteria [100%]	Deinococcus-Thermus [100%]	Deinococci [100%]	Thermales [98%]	Thermaceae [98%]	Meiothermus [98%]
**840**	Bacteria [100%]	Bacteroidetes [100%]	Bacteroidetes incertae sedis [95%]	Bacteroidetes incertae sedis [95%]	Bacteroidetes incertae sedis [95%]	Ohtaekwangia [95%]
**1007**	Bacteria [100%]	Acidobacteria [100%]	Acidobacteria Gp4 [100%]	—	—	—
**1306**	Bacteria [100%]	Deinococcus-Thermu [100%]	Deinococci [100%]	Thermales [97%]	Thermaceae [97%]	Meiothermus [96%]
**1366**	Bacteria [100%]	Acidobacteria [100%]	Acidobacteria Gp16 [100%]	—	—	—
**1464**	Bacteria [100%]	Proteobacteria [100%]	Alphaproteobacteria [98%]	—	—	—

**1716**	Bacteria [97%]	—	—	—	—	—
**1900**	Bacteria [100%]	—	—	—	—	—
**1950**	Bacteria [100%]	Actinobacteria [100%]	Actinobacteria [100%]	Actinomycetales [100%]	Geodermatophilaceae [94%]	Geodermatophilus [90%]
**2035**	Bacteria [100%]	Proteobacteria [100%]	Deltaproteobacteria [100%]	Myxococcales [100%]	Nannocystineae [100%]	—
**2109**	Bacteria [100%]	Proteobacteria [100%]	Alphaproteobacteria [100%]	Sphingomonadales [100%]	Sphingomonadaceae [87%]	—
**2143**	Bacteria [100%]	Proteobacteria [100%]	Betaproteobacteria [100%]	—	—	—
**2203**	Bacteria [100%]	Acidobacteria [100%]	Acidobacteria Gp13 [100%]	—	—	—
**2237**	Bacteria [100%]	Verrucomicrobia [100%]	—	—	—	—

**2253**	Bacteria [100%]	Proteobacteria [100%]	Alphaproteobacteria [100%]	Rhodobacterales [100%]	Rhodobacteraceae [100%]	__
**2257**	Bacteria [100%]	Proteobacteria [100%]	Betaproteobacteria [97%]	__	__	__
**2446**	Bacteria [100%]	Deinococcus-Thermus [100%]	Deinococci [100%]	Thermales [100%]	Thermaceae [100%]	__
**2582**	Bacteria [100%]	Proteobacteria [100%]	Alphaproteobacteria [100%]	Rhizobiales [99%]	__	__
**2774**	Bacteria [100%]	Proteobacteria [100%]	Betaproteobacteria [100%]	Rhodocyclales [95%]	Rhodocyclaceae [95%]	__
**3004**	Bacteria [100%]	Actinobacteria [97%]	Actinobacteria [97%]	__	__	__
**3047**	Bacteria [100%]	__	__	__	__	__
**3072**	Bacteria [100%]	Proteobacteria [100%]	Alphaproteobacteria [100%]	Rhizobiales [100%]	Bradyrhizobiaceae [100%]	Bradyrhizobium [100%]

**3089**	Bacteria [100%]	Chloroflexi [95%]	—	—	—	—
**3100**	Bacteria [100%]	Acidobacteria [100%]	Acidobacteria Gp4 [100%]	—	—	—
**3113**	Bacteria [100%]	Proteobacteria [100%]	Alphaproteobacteria [100%]	Caulobacteraceae [100%]	Caulobacterales [100%]	Phenylobacterium [100%]
**3357**	Bacteria [100%]	Proteobacteria [100%]	Alphaproteobacteria [100%]	Sphingomonadales [100%]	Sphingomonadaceae [100%]	Sphingomonas [89%]
**3607**	Bacteria [100%]	Gemmatimonadetes [95%]	Gemmatimonadetes [95%]	Gemmatimonadales [95%]	Gemmatimonadaceae [95%]	Gemmatimonas [95%]
**3730**	Bacteria [100%]	Acidobacteria [100%]	Acidobacteria Gp16 [100%]	—	—	—
**3824**	Bacteria [100%]	Proteobacteria [100%]	Alphaproteobacteria [100%]	Rhizobiales [100%]	—	—
**3837**	Bacteria [100%]	Acidobacteria [100%]	Acidobacteria Gp16 [100%]	—	—	—

**3843**	Bacteria [100%]	Actinobacteria [100%]	Actinobacteria [100%]	Acidimicrobiales [93%]	Acidimicrobineae [93%]	—
**3988**	Bacteria [100%]	Proteobacteria [100%]	Alphaproteobacteria [100%]	Rhodobacterales [100%]	Rhodobacteraceae [100%]	—
**4079**	Bacteria [100%]	Proteobacteria [100%]	Alphaproteobacteria [100%]	Sphingomonadales [100%]	Sphingomonadaceae [100%]	Novosphingobium [100%]
**4096**	Bacteria [100%]	Acidobacteria [100%]	Acidobacteria Gp16 [100%]	—	—	—
**4351**	Bacteria [100%]	Bacteroidetes [100%]	"Bacteroidetes" incertae sedis [88%]	"Bacteroidetes" incertae sedis [88%]	"Bacteroidetes" incertae sedis [88%]	Ohtaekwangia [88%]
**4388**	Bacteria [100%]	Actinobacteria [100%]	Actinobacteria [100%]	Actinomycetales [100%]	Mycobacteriaceae [100%]	Mycobacterium [100%]
**4461**	Bacteria [100%]	Deinococcus-Thermus [100%]	Deinococci [100%]	Thermales [99%]	Thermaceae [99%]	Meiothermus [99%]
**4798**	Bacteria [100%]	Bacteroidetes [100%]	Sphingobacteria [100%]	Sphingobacteriales [100%]	Chitinophagaceae [100%]	Flavisolibacter [97%]

**4863**	Bacteria [100%]	Deinococcus-Thermus [100%]	Deinococci [100%]	Thermales [100%]	Thermaceae [100%]	Meiothermus [100%]
**4873**	Bacteria [100%]	Deinococcus-Thermus [100%]	Deinococci [100%]	Thermales [100%]	Thermaceae [100%]	Meiothermus [100%]
**4923**	Bacteria [100%]	Bacteroidetes [100%]	Sphingobacteria [100%]	Sphingobacteriales [100%]	Chitinophagaceae [100%]	Flavisolibacter [100%]
**5002**	Bacteria [100%]	—	—	—	—	—
**5088**	Bacteria [100%]	Proteobacteria [100%]	Betaproteobacteria [99%]	—	—	—
**5118**	Bacteria [100%]	Proteobacteria [100%]	Alphaproteobacteria [100%]	Rhodobacterales [100%]	Rhodobacteraceae [100%]	—
**5295**	Bacteria [100%]	Actinobacteria [100%]	Actinomycetales [100%]	Micrococcineae [100%]	Microbacteriaceae [100%]	—
**5400**	Bacteria [100%]	Proteobacteria [100%]	Betaproteobacteria [100%]	—	—	—

**5415**	Bacteria [100%]	Proteobacteria [100%]	Alphaproteobacteria [100%]	Rhodobacterales [99%]	Rhodobacteraceae [99%]	—
**5436**	Bacteria [100%]	Actinobacteria [100%]	Actinobacteria [100%]	Actinomycetales [100%]	Microbacteriaceae [100%]	—
**5577**	Bacteria [100%]	Proteobacteria [100%]	Betaproteobacteria [100%]	Rhodocyclales [90%]	Rhodocyclaceae [90%]	—
**5699**	Bacteria [100%]	Proteobacteria [100%]	Alphaproteobacteria [100%]	Rhodospirillales [99%]	Acetobacteraceae [96%]	—
**5742**	Bacteria [100%]	Proteobacteria [100%]	Alphaproteobacteria [100%]	Rhodospirillales [100%]	Acetobacteraceae [100%]	—
**5874**	Bacteria [100%]	Proteobacteria [100%]	Alphaproteobacteria [100%]	Rhizobiales [100%]	—	—
