# Supplementary material for: Characterization of the core microbiota of the drainage and surrounding soil of a Brazilian copper mine
Source: Genet Mol Biol. 2015 Oct-Dec;38(4):484–9. doi: 10.1590/S1415-475738420150025 (PMC4763313; doi:10.1590/S1415-475738420150025)
Supplement: Table S3 - [file 1415-4757-gmb-S1415-475738420150025-s003.pdf]

Table S3. Core microbiota: OTUs present in all soil samples, and their taxonomic classification in the RDP database. The minimum cuttof value is 80%.

	RDP classifier [bootstrap]				
**OTU**	**Phylum**	**Class**	**Order**	**Family**	**Genus**
**310**	Proteobacteria [100%]	Betaproteobacteria [97%]	—	—	—
**2022**	Proteobacteria [100%]	Alphaproteobacteria [100%]	—	—	—
**2237**	Proteobacteria [100%]	Alphaproteobacteria [100%]	Sphingomonadales [100%]	—	—
**2299**	Actinobacteria [100%]	Actinobacteria [100%]	Acidimicrobiales [99%]	Acidimicrobineae [99%]	—
**2390**	Actinobacteria [100%]	Actinobacteria [100%]	Acidimicrobiales [99%]	Acidimicrobineae [99%]	—
**2756**	Acidobacteria [100%]	Acidobacteria Gp6 [100%]	—	—	—
**3072**	Proteobacteria [100%]	Alphaproteobacteria [100%]	Rhizobiales [100%]	Bradyrhizobiaceae [100%]	Bradyrhizobium [100%]
**3100**	Acidobacteria [100%]	Acidobacteria Gp4 [100%]	—	—	—
**3152**	Actinobacteria [100%]	Actinobacteria [100%]	Actinomycetales [100%]	—	—
**3730**	Acidobacteria [100%]	Acidobacteria Gp16 [100%]	—	—	—
**3824**	Proteobacteria [100%]	Alphaproteobacteria [100%]	Rhizobiales [100%]	—	—
**4143**	Actinobacteria [91%]	Actinobacteria [91%]	—	—	—

**4184**	Actinobacteria [100%]	Actinobacteria [100%]	Actinomycetales [100%]	—	—
**4342**	Actinobacteria [100%]	Actinobacteria [100%]	Actinomycetales [100%]	Geodermatophilaceae [99%]	Blastococcus [92%]
**5211**	Acidobacteria [100%]	Acidobacteria Gp6 [100%]	—	—	—
**5482**	Proteobacteria [100%]	Alphaproteobacteria [100%]	Rhizobiales [100%]	—	—
**5874**	Proteobacteria [100%]	Alphaproteobacteria [100%]	Rhizobiales [100%]	—	—
